# 1 alpha, 25-dihydroxylvitamin D3 promotes Bacillus Calmette-Guérin immunotherapy of bladder cancer

**DOI:** 10.18632/oncotarget.1494

**Published:** 2013-11-19

**Authors:** Jong-Wei Hsu, Peng-Nien Yin, Ronald Wood, James Messing, Edward Messing, Yi-Fen Lee

**Affiliations:** ^1^ Department of Urology, University of Rochester, Rochester, New York, USA; ^2^ Department of Pathology, University of Rochester, Rochester, New York, USA; ^3^ Department of Obstetrics and Gynecology, University of Rochester, Rochester, New York, USA; ^4^ Cornell University, Ithaca, New York USA

**Keywords:** vitamin D, Bacillus Calmette-Guérin, bladder cancer, immunotherapy, interleukin 8

## Abstract

Bacillus Calmette-Guérin (BCG), a vaccine against tuberculosis(TB), has been used and proven to be one of the most effective treatments for non-muscle invasive bladder cancer (BCa). However, the mechanisms of BCG action have not been completely understood, thereby limiting the improvement of BCG therapy. Vitamin D deficiency has been associated with a high risk of TB infection, and the beneficial effect of UV exposure in TB patients was proven to be mediated via activation of vitamin D signals of innate immune cells. Thus, vitamin D signals might be involved in mediating BCG immunotherapy. To test this hypothesis, we examined the impact of 1alpha, 25-dihydroxyvitamin D3 (1,25-VD) on BCG-induced response in BCa cells and macrophage cells. Our data revealed that 1,25-VD promotes BCG-induced interleukin 8 (IL-8) secretion by BCa cells, consequently inducing the migration of macrophage, THP-1. This THP-1 cell migration promoted by 1,25-VD can be blocked by IL-8 neutralized antibody. Furthermore, 1,25-VD increased BCG-induced expression of macrophage markers in THP-1 cell, and enhanced the BCG-induced THP-1 cytotoxicity against low-grade BCa cells. Importantly, a pre-clinical trial using the *N*-butyl-*N*-(4-hydroxybutyl)-nitrosamine (BBN)-induced BCa mouse model revealed that intravesical co-treatment of 1,25-VD with BCG can prolong mice survival. These data demonstrate a novel mechanism by which 1,25-VD promotes BCG-mediated anti-BCa pathways and provides a platform for improving BCG efficacy with combination of 1,25-VD.

## INTRODUCTION

Bladder cancer (BCa) is the fourth leading cancer in prevalence and the eleventh leading cancer cause of death in American men [1]. BCa ranks highest in cost from diagnosis to death per patient [2]. About 80% of BCa patients are diagnosed with non-muscle invasive tumors [3], which include carcinoma *in situ* (CIS); Ta stage (low-grade non-invasive); and T1 stage (invasiveness into lamina propria). For non-muscle invasive BCa, patients are usually treated with a transurethral resection of bladder tumor (TURBT) to remove the existing tumors. Then, patients undergo a series of intravesical therapies, such as Bacillus Calmette-Guérin (BCG) or mitomycin C, to eliminate the residual cancer cells and prevent recurrence.

BCG was initially developed as a vaccine for tuberculosis (TB) in 1921 and was used as an adjuvant immunotherapy for BCa in 1976 [4]. Since then, BCG has been the most effective adjuvant treatment for non-muscle invasive BCa. It was proven that BCG can prevent tumor recurrence and slow the progression to muscle invasive stage [5], thereby yielding a higher survival rate than TURBT surgery alone [6]. The mechanism of BCG in BCa therapy is known to involve both innate and adaptive immune cells. In brief, BCG is given intravesically, and the BCG adheres to urothelial cells through fibronectin [7] and alpha 5 beta 1 integrin receptors [8] followed by internalization. Urothelial cells are then induced to secrete cytokines, including IL-6, IL-8, and TNF-alpha, that recruit neutrophils and monocytes/macrophages [9]. Neutrophils play multiple roles in BCG therapy, through directly eliminating tumor cells by secreting TNF-related apoptosis-inducing ligand (TRAIL) [10] and indirectly contributing to tumor elimination by secreting cytokines to recruit effector cells, such as T cells and NK cells [5].

In addition to neutrophils, several lines of evidence suggest that macrophage actively mediates BCG-induced anti-BCa activity. Following BCG installation, increased numbers of macrophage, along with T cells, and natural killer (NK) cells are observed in BCa infiltrates. In addition to antigen presenting cells, macrophage also acts as a cytotoxic effector against BCa cells by releasing INF-alpha, INF-gamma, and NO after BCG stimulation [11]. Those effector molecules are known to induce cell apoptosis [11].

IL-8 is a potent chemoattractant for pro-inflammatory mediator, and it is expressed by immune cells and epithelial cells in response to BCG [12]. Urinary IL-8 can predict BCG responsiveness [13, 14]. Also, IL-2 secreted from BCG-stimulated macrophages contributes to the maturation of natural killer (NK) cells and cytotoxic T cells that serve as effector cells to kill bladder tumor cells [15]. BCG stimulates innate immune cells to secrete a panel of cytokine to further recruit adaptive immune cells, such as CD4+ and CD8+ T cells, as well as NK cells [16]. Then, Th1 cytokines are produced by both innate and adaptive immune cells, including IL-2, TNF-alpha, and INF-gamma, and these cytokines are required for eradicating tumor cells [5].

One recent study confirmed that the BCG-induced immune response in both innate and adaptive immune cells is critical for BCG efficacy where pre-existing BCG immunity improves BCG immune response to tumors in mice. The results were further proven in BCa patients showing that patients with pre-existing BCG response, determined by positive purified protein derivative (PPD) skin test, had a significantly better recurrence-free survival after standard BCG therapy than patients with a negative PPD skin test [17, 18].

The clinical BCG response rate in BC patients is 50-70%, and a significant amount of patients fail BCG therapy. Currently, there is no biomarker to predict the patient's BCG responsiveness. Also, the majority of BCG patients developed mild cystitis, including urgency, malaise, and fever [19]. Interestingly, many reports suggest BCG-induced symptoms are associated with therapeutic efficacy [5], including cytokine release in the urine [20] and cystitis symptoms. Occasionally, patients may suffer from severe side effects such as a life-threatening sepsis. Once severe symptoms occur, BCG therapy may be postponed or withheld, and patients need to take long-term anti-tuberculosis treatment to reduce the potential side effects [21]. Better understanding of BCG functional mechanism is indeed greatly needed for improving BCG therapy efficacy as well as development of biomarkers for a better selection of BCa patients before and during the therapy.

Accumulating evidence suggests that vitamin D is beneficial to BCG vaccination against TB. Low serum vitamin D level is associated with susceptibility to TB infection [22]. Recent studies found that activation of toll-like receptor 1/2 (TLR1/2) by mycobacteria, like BCG, activates vitamin D signals by up-regulation of the expression of vitamin D receptor (VDR) and CYP27 (the enzyme that converts 25-dihydroxyvitamin D to active 1,25-VD), thus leading to the induction of antimicrobial function [23]. VDR expression is detected in human superficial transitional cell carcinoma (TCC) of bladder [24]. Recent studies also indicated that low vitamin D level is associated with the risk of BCa [25], and high vitamin D plasma level protects against BCa. Therefore, it is likely that vitamin D signaling may contribute to BCa disease progression and response to BCG immunotherapy.

We examined the effects of 1,25-VD on BCG-stimulated cytokine production, macrophage infiltration, and the cytotoxicity against BCa exerted by infiltrated macrophage. Importantly, we performed a pre-clinical trial to test if 1,25-VD can promote BCG efficacy in a carcinogen-induced BCa mouse model.

## RESULTS

### 1,25-VD promotes IL-8 secretion by BCa cells

**Figure 1:**
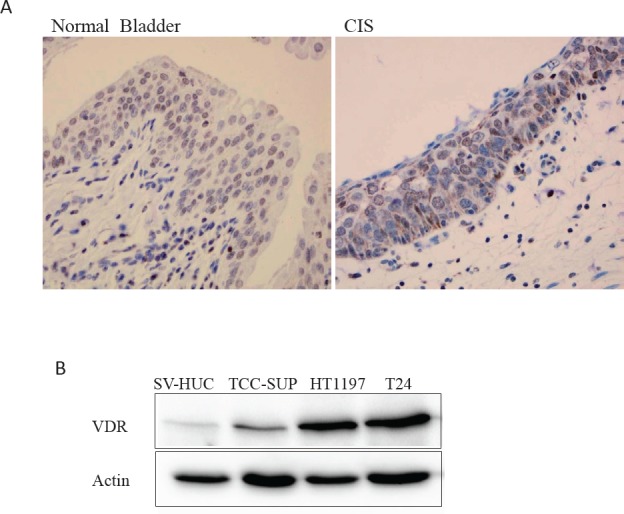
Detection of VDR expression in human bladder (A) VDR protein expression was detected in human bladder tissues including benign bladder and bladder carcinoma in situ (CIS) by IHC staining. (B) VDR expression in bladder cell lines, including immortalized normal urothelial cell line SV-HUC and three BCa cell lines, TCC-HUC (grade IV), HT1197 (low grade) and T24 (grade III) cells by Western blotting assay. β-actin was used as a loading control.

To understand if vitamin D signaling occurs in the human bladder, the expression and distribution of vitamin D receptor (VDR)-- the key factor that modulates vitamin D activity-- were examined in human bladder samples by immunohistochemistry (IHC) staining. As shown in Fig. 1A, we successfully detected VDR expression in both normal human urothelium and carcinoma *in situ* (CIS) so as to suggest a potential vitamin D action in the human bladder. Moreover, we also detected VDR expression levels in various bladder cell lines, including immortalized normal urothelial cell line; SV-HUC; and two high grade BCa cell lines; T24 (grade III); TCC-SUP (grade IV) and one low grade HT1197 by Western blot analysis. VDR is expressed in all the cells we examined with higher abundance in HT1197 and T24 cells and much less expressed in SV-HUC cells (Fig. 1B).

**Figure 2:**
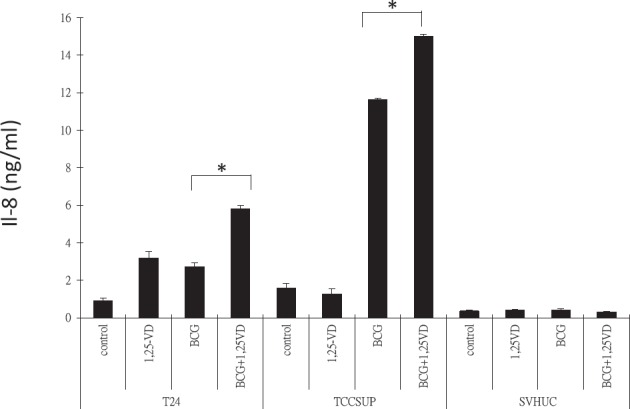
1,25-VD promotes BCG-induced pro-inflammatory IL-8 cytokine secretion 2 × 10^5^ TCC-SUP, T24, and SV-HUC cells were seeded in a 24-well culture plate in 10 % FBS RPMI medium. Cells were then treated with vehicle (ethanol), 100 nM 1,25-VD, 2 × 10^5^ CFU BCG, or in combination of 1,25-VD and BCG. Culture media were replaced with fresh media after 2 hours treatment of drugs, and cells were then cultured for another 22 hours. The supernatants were collected for assaying IL-8 pro-inflammatory cytokine secretion by ELISA (eBioscience).

Following BCG instillation, urothelial cells secrete a specific profile of pro-inflammatory cytokines, including IL-6, IL-8, and TNF-alpha [28, 29], which induce the first wave of immune response, innate immune cell infiltration. To test if 1,25-VD influences BCa cells cytokine production, we examined the levels of IL-6, IL-8, and TNF-alpha in BCa cells, TCC-SUP and T24 in response to BCG and/or 1,25-VD; SV-HUC immortalized cells were used as a control. As shown in Figure 2, BCG induced significant IL-8 production, and 1,25-VD can promote further the BCG-induced IL-8 secretion in both BCa cell lines. In contrast, the immortalized SV-HUC cells have very limited cytokine secretion in response to BCG and/or 1,25-VD (Fig. 2). Contrary to IL-8, we found 1,25-VD has very little influence on the BCG-induced IL-6 and TNF-alpha secretion (supplement data, Figs. 1A and 1B) in most of the cell lines we tested.

### 1,25-VD promotes BCG-induced macrophage migration via increasing Il-8 secretion

**Figure 3:**
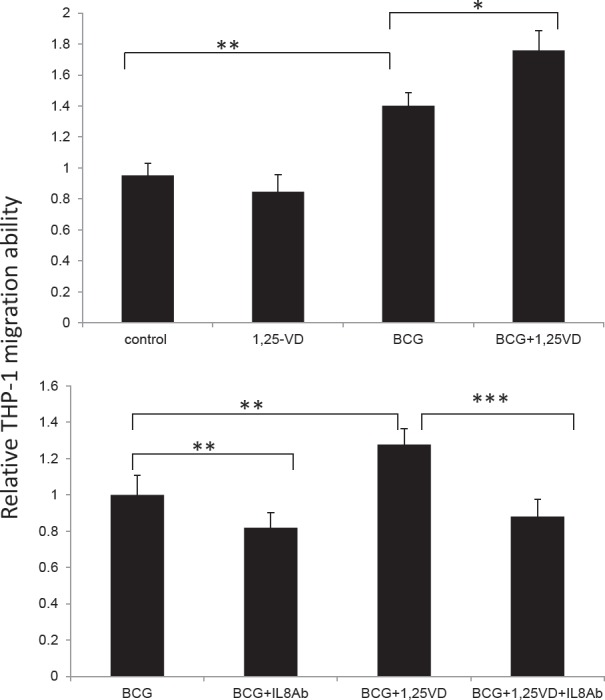
1,25-VD promotes THP-1 cell migration through IL-8 (A) 1,25-VD promotes BCG induced THP-1 cell migration by a transwell migration assay. TCC-SUP cells were treated with vehicle (ethanol), 100 nM 1,25-VD, 2 × 10^5^ CFU BCG, or in combination of 1,25-VD and BCG. Culture media were replaced with fresh media after 2 hours' treatment of drugs, then cells were cultured for another 22 hours. The conditioned media were collected and loaded in the lower chamber, and Thp-1 cells after serum-free starvation were added to transwell insert with 5 μm pore. After 24 hours the infiltrating THP-1 cells in the lower chamber were collected and counted. (B) Blockage of IL-8 by neutralized antibody reverses 1,25-VD-induced THP-1 migration. A transwell migration assay was performed as described in A, except adding IL-8 neutralizing antibody (20 μg/ml) with BCG and BCG+1,25-VD treated TCC-SUP cells. For statistical analysis, differences in mean values between two groups were analyzed by two-tailed Student's test. Results are the mean ± SEM of three experiments.

The pro-inflammatory cytokine secretion by BCa cells following BCG instillation would consequently induce innate immune cells infiltration into the bladder wall, and this first wave immune response is required for a successful BCG therapy [30]. Therefore, the 1,25-VD induction of IL-8 secretion by BCa cells could then promote BCG-induced innate immune cell migration. To test this, we performed a transwell migration assay whereby we treated TCC-SUP cells with vehicle control, 1,25-VD, BCG, and BCG+1,25-VD for two hours, then replaced with culture media for 22 hours incubation. Afterwards, the conditioned media that served as chemo-attractant sources were collected for migration of monocyte/macrophage THP-1 cells. We found that BCG treatment induced more THP-1 cell migration than vehicle control. Importantly, BCG+1,25-VD treatment induced statistically more THP-1 migration than BCG alone (Fig. 3 upper panel).

To confirm whether the THP-1 cell migration induced by BCG+12,5-VD treatment was due to the elevated IL-8, IL-8 neutralizing antibody that blocks IL-8 action was employed. As expected, we found that IL-8 neutralizing antibody significantly decreased both BCG- and BCG+1,25-VD-induced THP cell migration (Fig. 3, lower panel). It was noted that IL-8 neutralizing antibody can suppress both BCG and BCG+1,25-VD induced THP-1 migration to similar levels, suggesting Il-8 is the key pro-inflammatory cytokine that is involved in the THP-1 migration to BCa cells.

### 1,25-VD increased the expression of activated macrophage markers and inflammatory IL-8 expression

**Figure 4:**
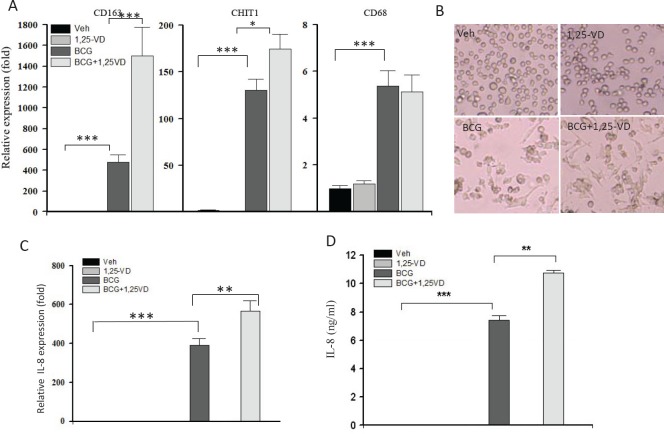
1,25-VD promotes BCG induced THP-1 differentation/maturation (A) THP-1 cells were treated with vehicle, 1.25-VD, BCG, or combination overnight. Cells were collected for total RNA extraction. The gene expression levels of macrophage markers, CD163, CHIT1, and CH68 were determined by real-time PCR assay. (B) Representative photos display a morphological alteration after treatments were shown. (C) The expression of IL-8 after the treatment was detected by the real-time PCR and ELISA assays.

Vitamin D signaling is known to be critical for controlling mycobacteria infection through the regulation of TLR signal in innate immune cells, such as macrophage. Given the critical roles of macrophage in initiating an effective response to BCG therapy, we determined the expression of activated macrophage markers and cytokine secretion in THP-1 cells in responses to BCG and/or 1,25-VD. Three activated macrophage markers, CD163, Chitotriosidase (CHIT) and CD68, were examined when THP-1 cells were treated with BCG and/or 1,25-VD. We found that all three macrophage markers were significantly induced by BCG treatment. CD163 and CHIT, but not CD68, expression levels were further enhanced by additional 1,25-VD treatment (Fig. 4) in THP-1 cells. With respect to the elevated macrophage marker expression, BCG also induces morphological alteration in THP-1 cells where the majority of BGC-treated THP-1 cells were attached with a spindle-like morphology as compared to undifferentiated state where cells are round and suspended as shown in vehicle treated cells. Importantly, we found more spindle-like cells were attached suggesting 1,25-VD promotes BCG-induced macrophage differentiation; unexpectedly, we did not see too much alteration in the 1,25-VD treated THP-1 cells.

Among those markers, CD163 was particularly interesting; it was induced significantly in the 1,25-VD+BCG group. CD163 is a cell surface receptor for bacteria and functions as an innate immune sensor for bacteria. The recognition of bacteria by CD163 could enhance inflammatory cytokine production in THP-1 cells [31]. Therefore, we tested if the combination of 1,25-VD with BCG could further induce cytokine secretion by THP-1 cells. Since we had shown in Figure 2 that 1,25-VD enhanced BCG-induced IL-8 production in BCa cells, we now tested if 1,25-VD can further promote IL-8 production in THP-1. As shown in Fig. 4B, BCG increased IL-8 expression at both mRNA and protein levels, and this induction can be further enhanced by 1,25-VD. These data suggested that 1,25-VD promotes macrophage maturation and activity in response to BCG.

### 1,25-VD promotes BCG-induced THP-1 cytotoxicity against BCa cells

**Figure 5:**
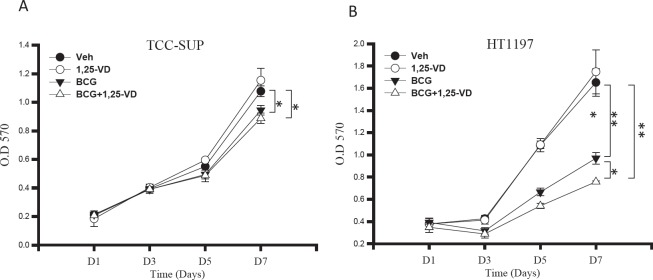
BCG-induced macrophage cytotoxcity against BCa cells THP-1 cells were treated with vehicle control, 1,25-VD, BCG and in combination for 24 hours. The cell supernatants were collected and analyzed for their cytotoxicity against TCC-SUP BCa cells (A) and HT-1197 BCa cells (B). Cell viability was determined by 7-day MTT assay. For statistical analysis, differences in mean values between the two groups were analyzed by two-tailed Student's test. Results are the mean ± SEM of three experiments.

It has been reported that BCG can induce macrophage cytotoxicity against BCa cells [11, 32]. As we showed, 1,25-VD can promote BCG-induced macrophage activation and cytokine production. We therefore tested if 1,25-VD can promote BCG induced macrophage cell cytotoxicity against BCa cells. We treated THP-1 cells with BCG and/or 1,25-VD, and conditioned media were collected for testing cytotoxicity against BCa cells. Two BCa cell lines were used: TCC-SUP and HT1197. As expected, BCG-treated THP-1 conditioned medium inhibited both TCC-SUP and HT1197 BCa cells growth significantly. Interestingly, 1,25-VD increased the BCG-mediated macrophage cytotoxicity against low-grade HT1197 cells but it only slightly increased cytotoxicity against high-grade TCC-SUP cells (Fig. 5). This cell specific 1,25-VD impact on BCG-induced macrophage cytotoxicity against BCa might be due to the lack of an optimal cell environment for 1,25-VD action, and low abundance of VDR expression in TCC-SUP cells (Fig. 1) as compared to HT1197 cells might be associated with less 1,25-VD impact on BCG-induced macrophage cytotoxicity against BCa cells.

### BCG and 1,25-VD prolongs survival in BBN induced BCa mice

Our *in vitro* results suggest that 1,25-VD promotes BCG-induced innate immune responses and recruits more macrophage cells toward BCa cells, thereby suppressing BCa cell growth. To further examine whether the BCG and 1,25-VD combination treatment can improve BCG efficacy *in vivo*, a carcinogen (BBN)-induced BCa mouse model was used [33]. BBN is a DNA damaging carcinogen derived from tobacco smoke. It induces BCa arising from urothelium in mice and represents a slowly progressing BCa mouse model in which mice develop an early pre-cancerous lesion that is similar to CIS (carcinoma *in situ*) in BCa patients. A total of 24 mice were given BBN in their drinking water to induce BCa. Following a 12-week BBN treatment, mice were randomly grouped into four treatments: vehicle control (100% ethanol); 1,25-VD (100 nM); BCG (1x10^6^ CFU); or BCG+1,25 for 6 weeks, while mice survival was monitored and recorded.

**Figure 6:**
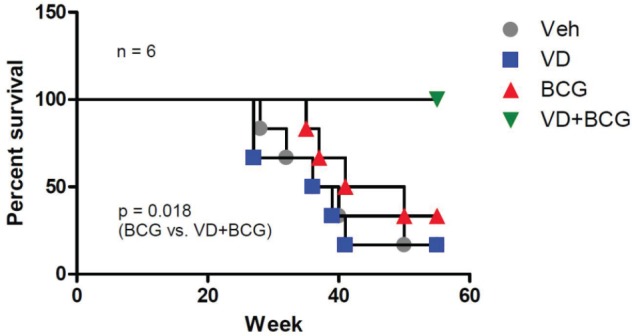
BCG+1,25-VD increases the survival rate of BBN-induced BCa mice 24 FVB female mice were initially given drinking water containing BBN at 6 weeks of age for 12 weeks' treatment. Subsequent to BBN treatment, 6 mice per group were subjected weekly to intravesical instillation of 100 μl PBS containing vehicle (ethanol), 100 nM 1,25-VD, 1 ×10^6^ CFU BCG, or 100 nM 1,25-VD plus 1 ×10^6^ CFU BCG. Mouse survival data were recorded weekly. The difference in survival rate was analyzed by Log-rank (Mantel-Cox) test.

The results showed that the BCG-treated group had only a slightly better overall survival rate than the control or 1,25-VD treated groups. Importantly, the combination of 1,25-VD with BCG greatly improved overall mice survival. The dead mice were found to have enlarged kidneys with hydronephrosis and non-transparent bladder tumor mass indicating that death was attributed to the BCa-induced obstruction of the urinary tracts. This pre-clinical data supports that combination of BCG with 1,25-VD results in better treatment efficacy than single drug treatment. Further determination of optimal dosages for BCG and 1,25-VD is highly desirable (Fig. 6).

## DISCUSSION

Combination therapy in which multiple drugs targeting the same cellular pathways could function synergistically for higher therapeutic efficacy has been used in clinics. In this current study, we demonstrated that BCG and 1,25-VD combination therapy leads to a better treatment efficacy than mono-therapy, which is clinically significant. First, a significant number of BCa patients fail respond to BCG, and BCG therapy commonly triggers local irritation effects and in rare cases, systemic toxicity. Second, the major limitation of systemic administration of pharmacologic doses of 1,25-VD is hypercalcemia side effect. Thus, this intravesical instillation of BCG and 1,25-VD combination therapy offers a unique opportunity to improve current BCG immunotherapy while avoiding the side effects caused by a single regimen at high concentration. In our study, we found that 1,25-VD can promote BCG-induced immune response; thereby, possibly, we can lower BCG concentration to reduce BCG-associated side effects without losing its potency by co-administration of 1,25-VD. Importantly, the intravesical administration of 1,25-VD provides the unique advantage that allows the use of high 1,25-VD concentration locally while avoiding hypercalcemia induced by high doses of 1,25-VD given systemically. In this current study, we chose to combine 1,25-VD at high concentration 100 nm with 1x10^6^ CFU BCG, the lower end of BCG concentration used in most animal studies [30, 34-36]. We showed that the combination treatment indeed has better efficacy than BCG alone. We did not observe any obvious toxicity or weight loss in the combination treated mice. This pre-clinical study suggests that we can reduce BCG concentration without compromising its anti-tumor effects by combining it with a higher dosage of 1,25-VD intravesically, through the mechanism involved in either enhancing innate immune responses [23] or antitumor effects [37]. A thorough pre-clinical study to determine the optimal combination doses is of great interest.

Even though we chose a lower concentration of BCG (10^6^ CFU) in our study, we expected to see BCG's anti-tumor effect. However, in this BBN-BCa mouse model, BCG treatment can only slightly improve mice survival. In contrast to MB49 syngeneic model, one of the most common mouse models used for BCG therapy study, 10^−6^ to 10^−7^ CFU BCG can improve MB-49 BCa mice survival [38]. One potential explanation for this reduced BCG efficacy might be associated with less availability of free fibronectin in this BBN-BCa model in which BCG was given at a very early stage of disease; fewer pre-cancer/cancer lesions are present, thereby less extracellular matrix fibronectins can be released. In contrast to the MB49 mouse model, the mice bladder walls are wounded first to allow MB49 BCa cells to adhere. This wounding process might also facilitate the fibronectin release. Interestingly, 1,25-VD was reported to increase fibronectin expression through a transcriptional regulation [39], which could then facilitate BCG internalization and yield better treatment efficacy.

IL-8 is a key prognostic marker for BCG immunotherapy for BCa [14, 20, 40-42]. A clinical study reported that patients who have detectable IL-8 in their urine after intravesical BCG treatment have a significantly higher disease-free survival [13]. Our data shows an increased IL-8 level induced by BCG and 1,25-VD combination therapy, thus enhancing the innate immune cell recruitment that promotes therapeutic efficacy. This result implies that a higher level of IL-8 in the bladder microenvironment is critical for BCG treatment. Therefore, to retain IL-8, for a longer period in the bladder potentially could be a strategy to improve BCG therapy. Previously, Zaharoff *et al* [35] demonstrated that intravesical instillation of chitosan and IL-12 exhibited a better therapeutic effect than BCG only, in which chitosan serves as an adhesive adjuvant and IL-12 as an immunotherapeutic agent with high potential to cure cancers [43]. Chitosan is cationic-charged and posses mucoadhesive properties, which enhance the adhesion of drug to negatively-charged bladder mucosa. In addition, its high viscosity can also enhance drug retention and promote drug permeability into urothelium by loosening the epithelial gap junctions [44]. Therefore, a combination of chitosan with Il-8 intravesical treatment could be a potential combination immunotherapy that needs further investigation.

The regulation of IL-8 production by vitamin D may vary under different physiological and pathological conditions. Our previous study suggested that 1,25-VD can suppress IL-8 production of prostate cancer cells via blocking NF-kappaB activation [45]. However, in this current study we found that 1,25-VD promotes IL-8 production only in the presence of BGC, suggesting this 1,25-VD promoting IL-8 is indirectly mediated through BCG-induced pathway. It is known that BCG binds to TLRs [46], which then induce the expression of an array of inflammatory cytokine genes, including IL-8 [47]. This activation of TLR signal by BCG could also activate vitamin D signal by enhancing the expression of VDR and CYP27beta1, and then inducing vitamin D downstream target expression such as anti-bacterial cathelicidins [48]. It is reported that cathelicidins, such as LL-37, can induce IL-8 production [49]. Therefore, the induction of IL-8 by co-treating 1,25-VD could be mediated indirectly via the activation of BCG-TLR downstream targets.

In summary, our study demonstrated a novel mechanism by which 1,25-VD promotes BCG-induced anti-BCa activity and this combination of BCG with 1,25-VD provides a platform for improving BCG efficacy.

## MATERIALS AND METHODS

### Cell lines and reagents

Human urothelial immortalized cell line SV-HUC, low grade BCa cells HT1197 and high grade BCa cells TCC-SUP (Grade IV), T24 (Grade III) based on SRCCM alignment [26] and human monocyte cell line THP-1 were purchased from the American Type Culture Collection. Human neutrophil-like cell line HL-60 was a gift from Dr. Nazzarreno Ballatori at the University of Rochester. All the cell lines were cultured in RPMI-1640 medium with 10% heat-inactivated fetal bovine serum and L-glutamine (10 mM; Gibco). BCG was purchased from Sanofi Pasteur. 1,25-VD was purchased from Sigma Aldrich.

### Western blot assay

Western blotting analysis was performed as previously described [27]. Briefly, cell lysate was prepared from each cell sample, and equal amounts of protein were resolved by electrophoresis in 10% or 15% SDS-PAGE gels and transferred to polyvinylidene difluoride membrane (Millipore, Bedford, MA). Antibodies used are human VDR (N-20; Santa Cruz Biotechnology, Santa Cruz, CA), and beta-actin (Santa Cruz Biotechnology, Santa Cruz, CA).

### ELISA assay

2×10^5^ BCa cells or THP-1 cells were seeded in each well of a 24-well plate. The cells were treated with vehicle, 100 nM 1,25-VD, 2×10^6^ CFU/mL BCG, or a combination of 100 nM 1,25-VD and 2×10^6^ CFU/mL BCG. After 24 hours of treatment, the conditioned medium were harvested, filtered through a 0.22 μm filter, and stored in −80°C for determination of pro-inflammatory cytokine levels including IL-8, IL-6, and TNF-alpha by ELISA assay (eBioScience).

### Transwell migration assay

Migration of macrophage was examined using a transwell assay. Thp-1 cells were cultured in serum free RPMI medium overnight, then 5×10^5^cells were added to each transwell insert with pore size 5 μm (Corning Inc.). The lower chambers, serving as the chemo-attractant source, were loaded with 500 μl conditioned media collected from the TCC-SUP cells treated with vehicle, 100 nM 1,25-VD, 5×10^5^ CFU BCG, or combination of BCG and 1,25-VD. Conditioned media were prepared by exposing the TCC-SUP cells to two hours of drug treatment, withdrawing the media and replacing them with fresh RPMI containing 10% FBS and incubating for 22 hours. After 24 hours, the infiltrating cells in the lower chamber were collected and counted. 10 μg/ml IgG or anti-human IL-8 neutralizing antibody (R&D systems) was added in the lower chamber to neutralize the IL-8 function in cell migration.

### Cytotoxicity assay

HT1197 and TCC-SUP cells were seeded in 96-well culture plates. Cells were treated with diluted conditioned medium derived from supernatant of BCG and/or 1,25-VD treated-THP-1 cells. Cell growth rate was determined by MTT assay. Briefly, culture media were replaced with fresh DMEM medium containing MTT stock solution and incubated at 370c for two hours. Medium was then removed, dissolving solvent DMSO was added, and absorbance was measured at a wavelength of 570 nm with background subtraction at 660 nm.

### RNA isolation and quantitative polymerase chain reaction (qPCR)

Total RNA was isolated with Trizol (Invitrogen). Reverse transcription was performed with iScript Master Mix (BioRad). Quantitative RT-PCR was performed as previously described using iQ™ SYBR® Green Supermix with CFX96 real-time PCR detection system (Bio-Rad). Gene expression was analyzed by using CFX manager software normalizing the expression to two housekeeping genes, beta-actin and GAPDH. Primer design for CD68, CD163, CHIT-1, and IL-8 is from the PrimerBank (http://pga.mgh.harvard.edu/primerbank).

### BBN-induced BCa mouse model and intravesical therapy of 1,25-VD and BCG

All animal procedures were approved by the University Committee on Animal Resources of the University of Rochester and were in accordance with the National Institutes of Health guidelines. To generate a carcinogen-induced BCa model in mice, 24 FVB female mice at six weeks of age were given drinking water *ad libitum* containing 0.05 % BBN (TCI America, Portland, OR) twice a week for 12 weeks. After completing BBN treatment, mice were subjected to four treatments of intravesical instillation: (1) vehicle control (ethanol); (2) 100 μM 1,25-VD; (3) 10^6^ CFU BCG; or (4) 100 μM 1,25-VD combined with 10^6^ CFU BCG. The drugs remained in the bladder lumens for one hour while animals were kept under isoflurane anesthesia. Intravesical treatments were administered once a week for six weeks. The mice survival rate was monitored.

### Statistical analysis

Differences in mean values between the two (control) groups were analyzed by a two-tailed Student's T test. Differences in the survival rates of mice with BCa between the two groups were analyzed by a Log-rank (Mantel-Cox) test. P-values less than 0.05 were considered statistically significant.

## Supplementary Data


