# Supplementary material for: 1 alpha, 25-dihydroxylvitamin D3 promotes Bacillus Calmette-Guérin immunotherapy of bladder cancer
Source: Oncotarget. 2013 Nov 19;4(12):2397–406. doi: 10.18632/oncotarget.1494 (PMC3926835; doi:10.18632/oncotarget.1494)
Supplement: Supplementary file 1 [file oncotarget-04-2397-s001.pptx]

## Slide 1
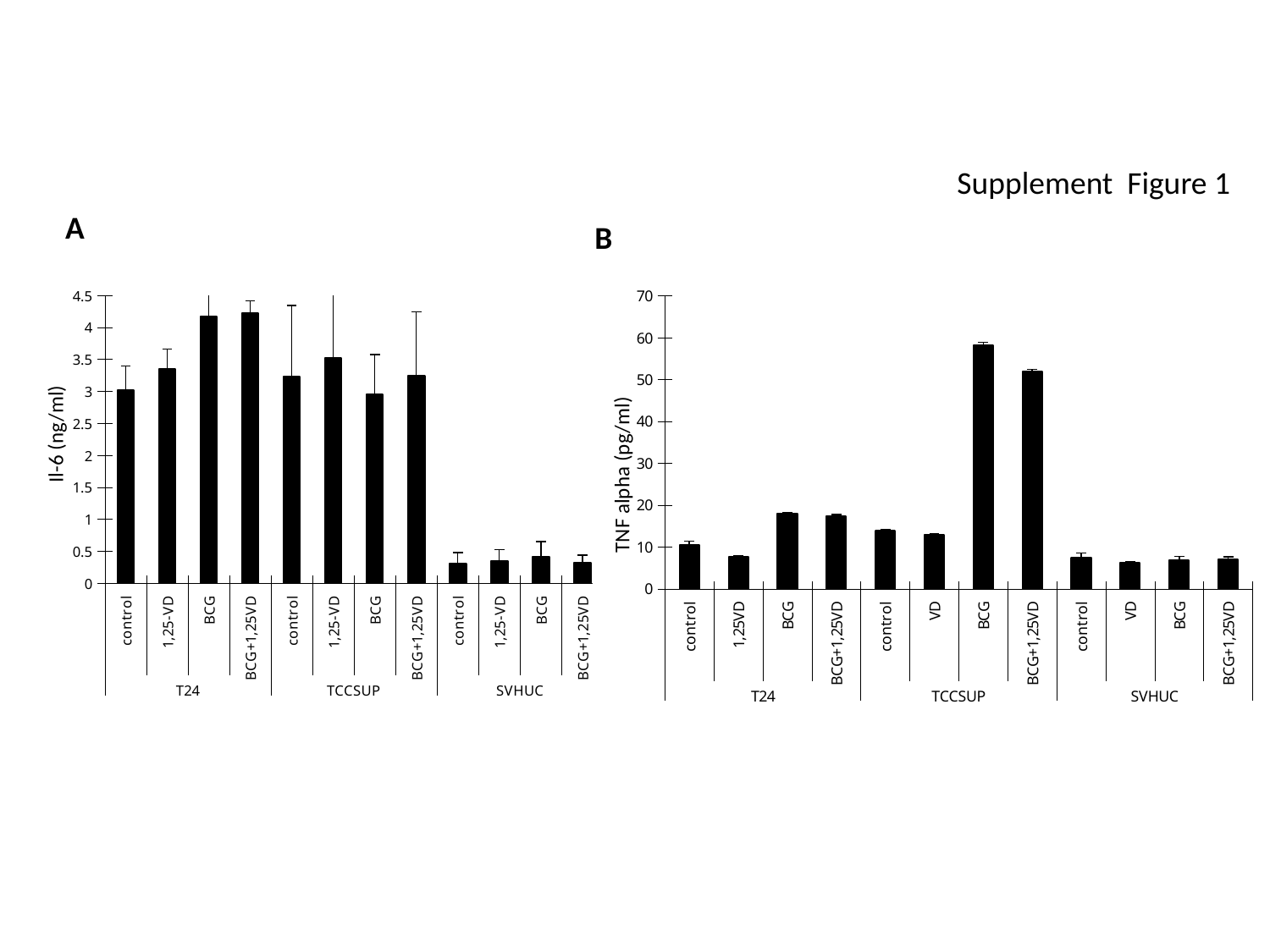

Supplement Figure 1
A
B
### Chart
| Category | |
|---|---|
| control | 3.0220732513518542 |
| 1,25-VD | 3.3549255175940726 |
| BCG | 4.170509615528332 |
| BCG+1,25VD | 4.225592714955001 |
| control | 3.2313264480274206 |
| 1,25-VD | 3.526434746875009 |
| BCG | 2.95318322287352 |
| BCG+1,25VD | 3.2412998447624197 |
| control | 0.30293825107351857 |
| 1,25-VD | 0.344818273795002 |
| BCG | 0.41224044274074084 |
| BCG+1,25VD | 0.31662440755500154 |
### Chart
| Category | |
|---|---|
| control | 10.377614000000024 |
| 1,25VD | 7.578191999999994 |
| BCG | 17.885445999999924 |
| BCG+1,25VD | 17.411402000000002 |
| control | 13.785606000000024 |
| VD | 12.843924000000001 |
| BCG | 58.23684000000001 |
| BCG+1,25VD | 51.92693000000001 |
| control | 7.51413199999998 |
| VD | 6.2649619999999855 |
| BCG | 6.841501999999995 |
| BCG+1,25VD | 7.033681999999999 |Il-6 (ng/ml)
TNF alpha (pg/ml)
